# Clinical and Therapeutic Notes

**Published:** 1887-12-17

**Authors:** C. R. Illingworth


### Clinical and Therapeutic Notes.

By C. R. Illingworth, M.D., M.R.C.S.

### Pyrexia and Anti-pyretics.

Any rise of the temperature of the body from the normal of 98·4 deg. indicates a febrile condition known as pyrexia. The increase varies in different diseases, and in the various stages of any given disease, from the normal to 103, 104, and up to 107 and 108 deg., beyond which point any further rise is incompatible with life.

Febrile phenomena are present both in purely inflammatory diseases and in diseases associated with a diminution of the fibrin-forming power. It is here to be noted though, that in the first instance there is a distinct increase in the fibrin elements of the blood in the septicæmic group, and particularly at the infective areas. Instances of this are found in the effusion of inflammatory lymph in the vicinity of the tonsils in scarlet fever, and near Peyer's patches in typhoid.

The cause of the pyrexia in the purely inflammatory class is the more rapid oxidation of the blood, and increased tissue change. Taking pneumonia as an example, we have a condition of *stasis* of the blood produced in the capillaries of the parenchyma of the lung, which by exciting the filaments of the vagus and sympathetic nerves leads to increase in the activity of the respiratory and circulatory centres. The result is a more rapid circulation throughout the whole of the systemic capillaries, with increased chemical change in the tissues. In the latter, or septic class of diseases, the liquefying action of the absorbed poison and developing germ will have the effect of still further irritating the circulatory centre, and exciting still greater chemical change in the tissues.

When a feverish condition tends to increase, and the thermometer registers a temperature of 106 deg., *hyperpyrexia* is said to exist. To check this development of heat certain methods of treatment are adopted and certain drugs administered, which are known as *antipyretic* in their effect upon the economy. The list of antipyretics is a large one. It includes aconite, digitalis, quinine, salicine, salicylate of soda, diaphoretics, and diuretics, blood-letting, ice-packs, cold baths, cold sponging, cold air, hot baths, iced enemata, cold water coils, etc. Quite recently several new drugs have been found possessing great power in this direction. They are antipyrine, antifebrine, kairine, and thalline.

In this connexion a group of remedies should be noticed, which, from their supposed power of cooling the body in disease, and hence allaying febrile disturbance, are named *refrigerants*. It comprises water, acetic acid, citric acid, tartaric acid, cream of tartar in solution, phosphoric acid, nitrate of potash, chlorate of potash, grape juice, orange juice, lemon juice, and tamarinds. As regards the effects of these: "It will be observed that they differ very much among themselves, although most of them belong to the group of acid and astringent remedies; their action in lowering the temperature of the body has never been clinically established, and is doubtful; still it is a fact that, when a patient is feverish, the acids and the juices of acidulous fruits are very grateful n relieving thirst" (Garrod). It will thus be evident that the group of refrigerants is a much less clearly defined one than that of antipyretics, and, indeed, that in the main it has reference to those subjective phenomena in the patient, of cooling sensations and relief from urgent thirst; whilst the lowering of the temperature of the body by antipyretics is clearly established by the thermometer.

Although the fact of reduction cannot be doubted, one glance at the list of antipyretics is sufficient for the realisation of the widely different means by which this is effected. Thus aconite and digitalis by their depressant action upon the heart strike at the root of increased oxidation of the blood, by diminishing the rapidity of the circulation; salicylate of soda acts by preventing the formation of fibrin in common with all the salts of that base, and with redoubled intensity compared with any other, removes obstructions in the course of the circulation, and promotes a profuse diaphoresis by simple liquefaction; air baths, ice packs, and warm and cold baths act upon the skin, and further radiation and evaporation in that direction; whilst quinine and some others have a distinct, but as yet little understood, action upon the nervous system, and, in certain septic diseases, well-marked germicidal powers. The theory held concerning the most recently discovered antipyretics is that they act through the nerve centres; but a study of the untoward effects sometimes observed after their administration would seem to point in another direction. Antipyrine, the first of these artificial alkaloids, was discovered in 1884. It is one of the products of the destructive distillation of coal tar. Its formula is $C_{20}H_{18}N_4O$, and it can be prepared synthetically. It is soluble in water, and can be given in wine or syrup of orange, in doses of from ten to thirty grains. For the first three times it may be administered every hour, but after that at longer intervals. It has been said that "wherever you have a high temperature you have indications for the administration of antipyrine." The safety of this remains to be proved. A reduction of temperature is seen in the course of half an hour, and proceeds further and further, surely and steadily, for five or six hours. The higher the abnormal temperature, the more marked is the effect of this powerful drug upon it. Perspiration is generally induced. For a child the dosage is one and a-half grains for every year of its age.

There are certain contra-indications for the use of the drug which are (I think wisely) observed by some. These are urgent vomiting, profuse perspiration, and extreme cardiac weakness. There are one or two recorded cases of collapse from its use, but it has been urged that this accident has so rarely happened, that there is no need to consider it a danger. In fine, it is generally conceded that so long as an antipyretic is needed, so long may you continue to administer antipyrine.

*Antifebrine*, the second of this group, has the formula, $C_6H_5NHC_2H_3O$, and can also be prepared synthetically. The dose is from four to fifteen grains, and it may be given in water, wine, or wafers. Four grains are equivalent to sixteen of antipyrine, and from thirty to sixty grains may be administered in the twenty-four hours. Its special advantages are its prompt action, the slight perspiration it induces, and the fact that it does not produce rigors, as is sometimes the case with antipyrine.

*Kairine* has a similar antipyretic action, but its effects are not so pronounced and not so lasting. It sometimes produces rigors. The dose is from ten to fifteen grains. *Thalline*, the fourth, calls for no especial mention, and is little used. But, as has already been mentioned, untoward effects have sometimes been observed to follow the administration of these four antipyretics. These are cyanosis of the face and extremities, collapse, and in one or two instances, death. Certain investigations by Continental authorities have led to the opinion that there is an injurious effect exerted by all of them upon the red blood corpuscles. The production of cyanosis is an indication that there is some interference with the process of oxygenation of the blood, and consequently with the fibrin-forming power of that fluid. Here, therefore, it would seem, we have a contra-indication for the use of antipyretics of this nature in the septicæmic group of diseases. For the same reason remedies such as the salicylate of soda and carbonate of ammonia should never be prescribed in diseases associated with a diminished fibrin-forming power, whilst they are of signal service in those of a purely inflammatory nature, such as croupous pneumonia and acute nephritis.

